# Lon protease inactivation in *Drosophila* causes unfolded protein stress and inhibition of mitochondrial translation

**DOI:** 10.1038/s41420-018-0110-1

**Published:** 2018-10-22

**Authors:** Gautam Pareek, Ruth E. Thomas, Evelyn S. Vincow, David R. Morris, Leo J. Pallanck

**Affiliations:** 1 Department of Genome Sciences, University of Washington, 3720 15th Avenue NE, Seattle, WA 98195 USA; 2 Department of Biochemistry, University of Washington, 1705 NE Pacific St., Seattle, WA 98195 USA

## Abstract

Mitochondrial dysfunction is a frequent participant in common diseases and a principal suspect in aging. To combat mitochondrial dysfunction, eukaryotes have evolved a large repertoire of quality control mechanisms. One such mechanism involves the selective degradation of damaged or misfolded mitochondrial proteins by mitochondrial resident proteases, including proteases of the ATPase Associated with diverse cellular Activities (AAA^+^) family. The importance of the AAA^+^ family of mitochondrial proteases is exemplified by the fact that mutations that impair their functions cause a variety of human diseases, yet our knowledge of the cellular responses to their inactivation is limited. To address this matter, we created and characterized flies with complete or partial inactivation of the *Drosophila* matrix-localized AAA^+^ protease Lon. We found that a *Lon* null allele confers early larval lethality and that severely reducing Lon expression using RNAi results in shortened lifespan, locomotor impairment, and respiratory defects specific to respiratory chain complexes that contain mitochondrially encoded subunits. The respiratory chain defects of *Lon* knockdown (*Lon*^*KD*^) flies appeared to result from severely reduced translation of mitochondrially encoded genes. This translational defect was not a consequence of reduced mitochondrial transcription, as evidenced by the fact that mitochondrial transcripts were elevated in abundance in *Lon*^*KD*^ flies. Rather, the translational defect of *Lon*^*KD*^ flies appeared to be derived from sequestration of mitochondrially encoded transcripts in highly dense ribonucleoparticles. The translational defect of *Lon*^*KD*^ flies was also accompanied by a substantial increase in unfolded mitochondrial proteins. Together, our findings suggest that the accumulation of unfolded mitochondrial proteins triggers a stress response that culminates in the inhibition of mitochondrial translation. Our work provides a foundation to explore the underlying molecular mechanisms.

## Introduction

Mitochondria are responsible for most of the energy produced by a cell, but the generation of reactive oxygen species (ROS) as a byproduct of this activity can damage mitochondrial proteins, lipids, and DNA^1–3^. Also, while mitochondria contain their own genome, most mitochondrial proteins are encoded in the nucleus, and a stoichiometric imbalance between mitochondrial and nuclear encoded respiratory chain subunits can cause misfolding and aggregation of the unassembled proteins^4,5^. Fortunately, there are many surveillance pathways that oppose or reverse this damage, including the AAA^+^ family of mitochondrial proteases^6–8^. All of the AAA^+^ proteases form multimeric protein complexes and use ATP to unfold and transport substrates to an internal proteolytic cavity for degradation. In higher eukaryotes, there are five major mitochondrial AAA^+^ proteases that are distinguished by their subunit composition and mitochondrial localization. The most well-studied member of the AAA^+^ protease family is Lon.

Previous work indicates that Lon possesses three different activities, serving as a chaperone and DNA-binding protein in addition to its role in proteolysis^9,10^. Lon expression is regulated by multiple cellular stresses, including ROS and unfolded protein stress, and there is substantial support for the role of Lon in the degradation of oxidatively damaged and misfolded proteins^11,12^. Although few Lon substrates are known with certainty, a number of candidate Lon substrates have been identified from biochemical studies aimed at the identification of Lon-binding proteins, including the mitochondrial DNA (mtDNA) replication factors Twinkle, polymerase gamma, Tfam, and the chaperones Hsp60 and mtHsp70^13–19^. Subsequent studies aimed at validating the significance of these binding interactions indicated that Lon inactivation results in increased mtDNA copy number and destabilization of Hsp60 and mtHsp70 under environmental stress^20–22^. However, it is unclear whether these findings reflect direct effects of Lon inactivation, or downstream cellular responses to loss of Lon activity. Moreover, mutations in *Lon* have been shown to result in the recessive developmental disorder CODAS (cerebral, ocular, dental, auricular, and skeletal) syndrome, yet the mechanisms by which mutations in *Lon* cause this disease are currently unknown^23^.

To create a simple, genetically tractable model system to explore the biological role of Lon and the pathological consequences of Lon inactivation, we used CRISPR/Cas9-mediated gene targeting and RNAi to create *Drosophila* strains with complete and partial loss of Lon function. We found that *Lon* is an essential gene in *Drosophila* and that flies expressing RNAi against Lon (*Lon* knockdown flies, or *Lon*^*KD*^ flies) had shortened lifespan, defective locomotion, and altered respiratory chain activity. The respiratory chain deficits in *Lon*^*KD*^ flies were specific to respiratory chain complexes that contain subunits encoded by the mitochondrial genome, suggesting that altered expression of mitochondrially encoded components underlies this defect. Consistent with this conclusion, we found that reduced mitochondrial complex activity is accompanied by reduced complex abundance and diminished mitochondrial translation. The translational defect of *Lon*^*KD*^ flies was not a consequence of reduced mitochondrial transcription, but rather appeared to be a consequence of reduced association of mitochondrial transcripts on mitochondrial ribosomes and packaging of mitochondrial transcripts into highly dense ribonucleoparticles. The translational defect of *Lon*^*KD*^ flies was also accompanied by elevated abundance of unfolded mitochondrial proteins, and overexpression of another matrix protease, ClpP, partially rescued the defects associated with Lon inactivation, possibly by reducing the burden of unfolded mitochondrial proteins. Together, our findings strongly suggest that Lon inactivation results in the activation of an unfolded protein stress response that inhibits mitochondrial translation.

## Results

### Creation of Lon-deficient *Drosophila* strains

To explore the biological roles of Lon protease we used CRISPR/Cas9 technology to create a null allele of the *Drosophila Lon* gene (*CG8798)*. Briefly, we constructed guide RNAs designed to create double-strand breaks flanking the *Lon* coding sequence (Suppl. Figure 1). We also created a donor vector construct consisting of the *DsRed* marker flanked by 5′ and 3′ untranslated sequences from the *Lon* coding region for use in homology-directed recombinational repair of the double-strand breaks. Flies expressing the *DsRed* marker were then subjected to whole-genome sequencing to verify correct targeting of *DsRed* to the *Lon* locus and complete deletion of the *Lon* gene. Flies heterozygous for this deletion were fully viable with no detectable phenotypes. However, homozygotes died at the second instar larval stage of development, demonstrating that Lon is essential for viability (Suppl. Figure 2a).

**Fig. 1:**
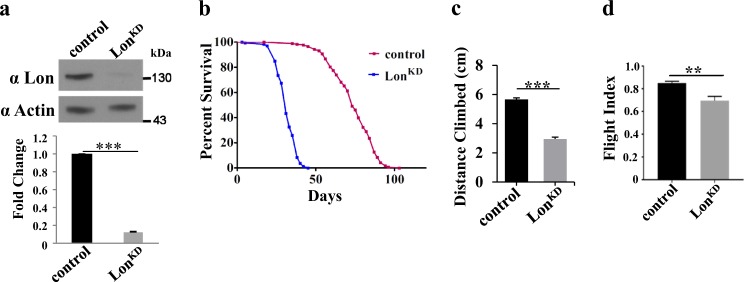
Inactivation of Lon results in shortened lifespan and defective locomotion. **a** Immunoblot analysis from heads of 1-day-old control and *Lon*^*KD*^ flies using Lon and actin antibodies. The Lon band intensity is normalized against actin as a loading control. Significance was determined using Student’s *t*-test (***p *<* 0.0005 by Student’s *t*-test). The experiment was repeated at least three times. **b** Kaplan–Meier survival curves of *Lon*^*KD*^ flies (*n* = 167, 50% survival 31 days) and controls (*n* = 174, 50% survival 73 days) (****p < 0.0001 by Mantel–Cox log-rank test). **c** One-day-old *Lon*^*KD*^ flies exhibit a climbing defect. Error bars represent SEM (*n* *=* 135 for *Lon*^*KD*^, 134 for control, ***p < 0.0005 by Student’s *t*-test). **d** One-day-old *Lon*^*KD*^ flies exhibit a significant decrease in flight index (*n* *=* 6 independent groups of 10–15 animals, **p < 0.005 from Student’s *t*-test). Control = *UAS-mCherry-RNAi* driven by *elav-GAL4* and *da-GAL4*. *Lon*^*KD*^ = *UAS-Lon-RNAi-1* driven by *elav-GAL4* and da-GAL4

RNAi often reduces but does not completely eliminate target gene expression. Thus, we tested whether RNAi lines targeting *Lon* would circumvent the lethality conferred by a null mutation in *Lon*. We tested two different RNAi constructs targeting *Lon* (designated *Lon-RNAi-1* and *Lon-RNAi-2*) that we used in previously published work to explore the influence of Lon on the abundance and activity of the mitophagy factor PINK1^24^. Our previous work demonstrated that these RNAi lines differed in the efficiency by which they reduced Lon expression, with the *Lon-RNAi-2* line resulting in greater reduction in Lon expression. We expressed each RNAi line using two *GAL4* drivers simultaneously: the pan-neuronal *elav*-*GAL4* driver and the ubiquitous *da-GAL4* driver. Driving the stronger *Lon-RNAi-2* line with this combination of *GAL4* drivers failed to yield viable adult flies, but flies expressing the weaker *Lon-RNAi-1* transgene were fully viable and fertile as young adults, and had no obvious morphological alterations. Western blot analyses performed on heads and whole flies indicated that Lon expression was significantly reduced in flies bearing the *UAS-Lon-RNAi-1*, *elav*-*GAL4*, and *da-GAL4* transgenes compared to controls expressing RNAi against the exogenous *mCherry* sequence (*UAS-mCherry-RNAi*) (Fig. 1a and Suppl. Figure 2b). Flies bearing the *Lon-RNAi-1*, *elav*-*GAL4*, and *da-GAL4* transgenes were used in most of the remaining studies and will hereafter be called *Lon*^*KD*^.

### Partial inactivation of Lon results in shortened lifespan, locomotor defects, and altered respiratory chain activity

To further characterize the *Lon*^*KD*^ phenotypes, we subjected the flies to several standard *Drosophila* behavioral assays. Although *Lon*^*KD*^ flies appeared normal upon eclosion, they were significantly shorter-lived than the control flies (Fig. 1b). The maximal and median lifespans of *Lon*^*KD*^ flies were similar to those of flies bearing null mutations in genes encoding the other AAA^+^ mitochondrial protease family members *dYME1L* and *SPG7*^25,26^. Young *Lon*^*KD*^ flies also exhibited defects in climbing and flight starting early in life (Fig. 1c, d).

**Fig. 2:**
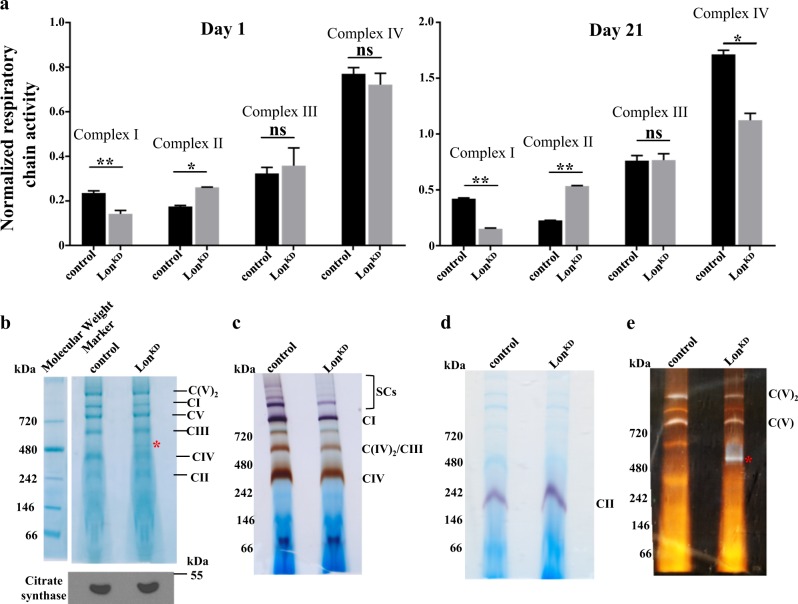
*Lon* knockdown flies have altered respiratory chain function and abundance. **a** The activity of respiratory chain complexes in control and *Lon*^*KD*^ flies at 1 day (left panel) and 21 days (right panel) of age (*n* = 2 independent groups of 500 adult flies, *p < 0.05, **p < 0.005 from Student’s *t*-test). **b** BN-PAGE analysis of mitochondrial protein extracts from 21-day-old control and *Lon*^*KD*^ flies. The red asterisk marks the location of the subcomplex containing F_1_ subunit of ATP synthase that is only detected in *Lon*^*KD*^ flies. Immunoblot of citrate synthase (bottom panel) was used as a loading control. **c** In-gel activity of mitochondrial complexes I and IV isolated from 21-day-old adults. SCs here refer to the supercomplexes. **d** In-gel activity of mitochondrial complex II isolated from 21-day-old flies. **e** In-gel activity of mitochondrial complex V isolated from 21-day-old adults. The red asterisk denotes the location of the subcomplex containing F_1 _subunit of ATP synthase in *Lon*^*KD*^ flies. Note that the protein molecular weight markers shown in (**b**) were used as reference to mark the gels in (**c–e**). For BN-PAGE and in-gel activity assays, the images shown are representative of two independent biological replicates. Control = *UAS-mCherry-RNAi* driven by *elav-GAL4* and *da-GAL4*. *Lon*^*KD*^ = *UAS-Lon-RNAi-1* driven by *elav-GAL4* and *da-GAL4*

Inactivation of Lon in other model systems results in reduced respiratory chain activity^27^. Thus, we tested whether *Lon*^*KD*^ flies exhibit similar respiratory chain defects, using established biochemical assays to quantify respiratory chain activity in young (1 day) and old (3 weeks) *Lon*^*KD*^ flies. We found that respiratory chain complexes I and IV had reduced activity in old *Lon*^*KD*^ flies compared to the controls, while young flies showed reduction only in complex I activity (Fig. 2a). However, complex II activity was increased significantly in both young and old *Lon*^*KD*^ flies. These alterations could result from a functional change in respiratory chain activity or from altered respiratory chain abundance. To distinguish between these possibilities while simultaneously confirming and extending our biochemical studies, we assessed the abundance of assembled complexes and their corresponding activities using blue native PAGE (BN-PAGE) and in-gel enzyme activity assays^28^. This work revealed that complexes I, III, and IV all exhibited both reduced activity and reduced abundance in *Lon*^*KD*^ flies, whereas complex II exhibited an increase in activity (Fig. 2b–d). Additionally, this work revealed the presence of a partially assembled but catalytically active F_1_ subunit–containing subcomplex of ATP synthase (complex V) (Fig. 2b, e). These alterations were also accompanied by a reduction in ATP content in *Lon*^*KD*^ flies (Suppl. Figure 3). Together, our findings indicate that partial inactivation of Lon results in a progressive age-dependent decline in oxidative phosphorylation capacity, and that this decline may underlie the shortened lifespan and behavioral deficits of *Lon*^*KD*^ flies.

### Lon knockdown results in increased mtDNA-encoded transcript abundance and reduced translation

All of the respiratory chain complexes exhibiting reduced abundance and assembly in *Lon*^*KD*^ flies (complexes I, III, IV, and V) contain one or more subunits encoded by mtDNA. Additionally, our BN-PAGE and in-gel activity assays in *Lon*^*KD*^ flies indicated that the ATP synthase F_1_ subunit containing subcomplex (consisting entirely of subunits encoded by nuclear DNA) accumulated at the expense of the F_O_ subcomplex (which includes two subunits encoded by mtDNA). By contrast, complex II, which exhibited increased activity in *Lon*^*KD*^ flies, consists entirely of nuclear DNA–encoded components. These findings led us to hypothesize that reduced mtDNA abundance, reduced transcription of mtDNA-encoded subunits, and/or reduced translation of mitochondrial transcripts might account for the decreased expression of complexes I, III, IV, and V.

**Fig. 3:**
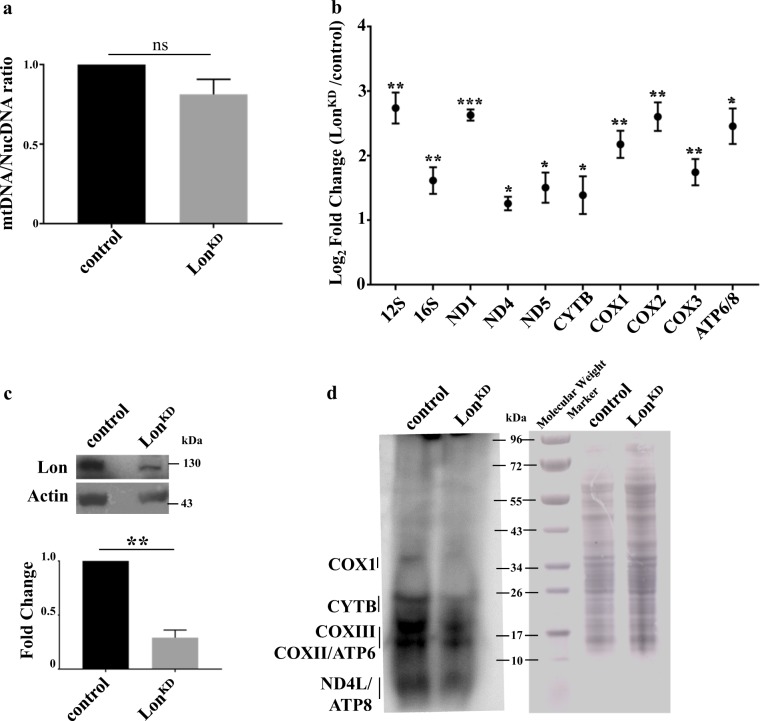
*Lon* knockdown flies exhibit increased mitochondrial transcript abundance and decreased mitochondrial translation. **a** Mitochondrial DNA abundance was compared in *Lon*^*KD*^ and control flies using qPCR to compare the ratio of mtDNA-encoded *mt:Cyt-b* to that of nuclear-encoded *Act79b* (*n* = 3 independent groups of 40–45 fly heads). Statistical significance was determined using Student’s *t*-test. **b** qRT-PCR was used to quantify steady-state abundance of the indicated mitochondrial RNAs in 21-day-old adult fly heads. Mitochondrial RNA abundance was normalized to the abundance of the nuclear-encoded *Act79b* transcript (*n* = 3 independent groups of 40–45 fly heads). Error bars indicate mean ± SEM. Student’s *t*-test was applied, *p < 0.05, **p < 0.005, ***p < 0.0005. **c** Immunoblot analysis of third instar larvae to confirm knockdown of Lon using actin as a loading control. *n* = 3 independent groups of five third instar larvae. Significance was determined using Student’s *t*-test, **p < 0.005. **d** *In organello* translation was performed using mitochondria isolated from third instar larvae. Mitochondria were labeled by incubating with ^35^S-methionine for 1 h. Positions of individual mitochondrially encoded proteins are indicated (left panel). Coomassie-stained gel (right panel) was used as a loading control. Control = *UAS-mCherry-RNAi* driven by *elav-GAL4* and *da-GAL4*. *Lon*^*KD*^ = *UAS-Lon-RNAi-1* driven by *elav-GAL4* and *da-GAL4*

To begin to explore these hypotheses, we first compared mtDNA abundance in *Lon*^*KD*^ flies and the controls. While increased mtDNA abundance in response to Lon inactivation has been reported^20^, mtDNA copy number was unchanged in *Lon*^*KD*^ flies (Fig. 3a). This is consistent with our previous finding of unchanged mtDNA abundance when Lon was knocked down with *elav-GAL4* alone^24^. We next analyzed the steady-state levels of several mitochondrial transcripts by qRT-PCR. We found that levels of all transcripts analyzed, including *12S* and *16S* rRNA and several mRNAs, were increased in old *Lon*^*KD*^ flies (Fig. 3b). Additionally, the transcript abundance of the mitochondrial transcription-promoting factors *TFAM* and *mtTFB2* was unchanged in *Lon*^*KD*^ flies (Suppl. Figure 4). Together, these findings indicate that the respiratory chain defects in *Lon*^*KD*^ flies do not derive from reduced mitochondrial gene dosage or reduced mitochondrial transcription.

To test whether *Lon*^*KD*^ flies manifest a translational defect, we used ^35^S-labeled methionine to perform *in organello* labeling of mitochondria prepared from adult flies. However, we detected very little labeling of mitochondrial proteins in our control flies (Suppl. Figure 5). This finding may reflect the fact that mitochondrial proteins are extremely long-lived^29^ and that there is thus a decreased need for mitochondrial translation during the adult stage of development. However, previous work has detected robust labeling of mitochondrial proteins using mitochondria obtained from larvae, so we repeated our *in organello* labeling studies using mitochondria from third instar larvae^30^. Our western blot analysis confirmed that Lon expression was greatly reduced in *Lon*^*KD*^ larvae relative to the controls (Fig. 3c). This experiment revealed a substantial decrease in de novo labeling of mitochondrial translation products in *Lon*^*KD*^ larvae relative to the controls (Fig. 3d), suggesting that the decreased abundance of complexes I, III, IV, and V in *Lon*^*KD*^ flies is a direct consequence of a translational defect. The increased abundance of mtDNA-encoded transcripts and nuclear DNA–encoded complex II may represent compensatory responses to alleviate this translational deficiency^31–33^.

### Mitochondrial transcripts are packaged into untranslated particles in Lon^KD^ flies

**Fig. 4:**
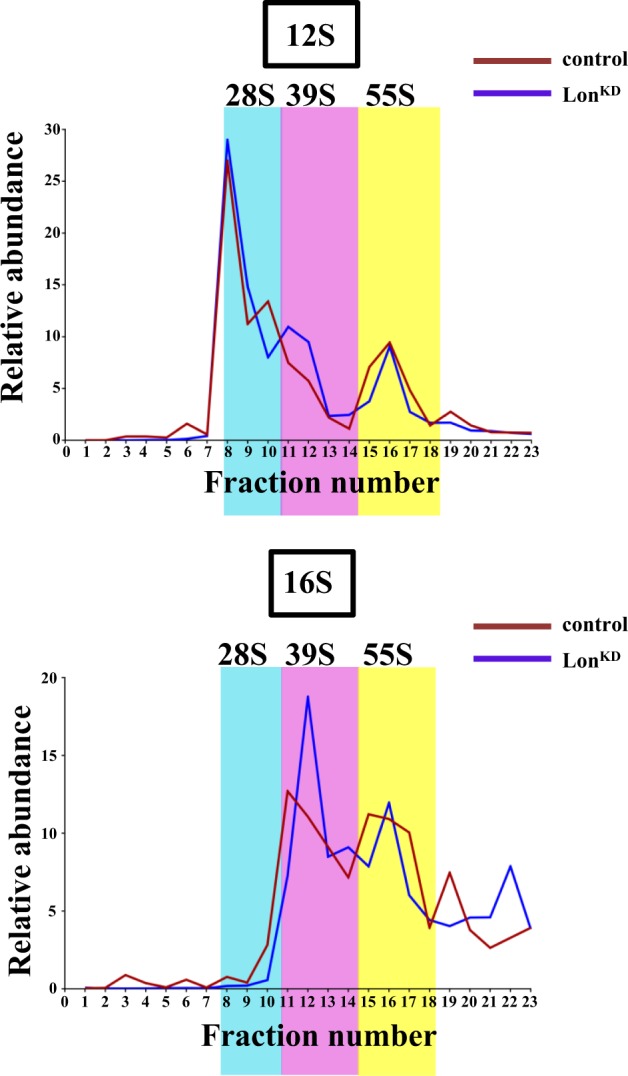
Mitochondrial ribosome assembly is only mildly affected in *Lon*^*KD*^ flies. Mitochondrial lysates from 21-day-old control and *Lon*^*KD*^ flies were subjected to sucrose density gradient fractionation to assess the state of assembly of mitochondrial ribosomes. The relative proportions of the small (28S) subunit, large (39S) subunit, and fully assembled (55S) mitochondrial ribosomes were assessed by subjecting the density gradient fractions to qRT-PCR using primer sets specific to *12S* rRNA, which marks the small subunit, and to *16S* rRNA, which marks the large subunit. Co-localization of the *12S* and *16S* rRNAs is diagnostic of fully assembled and actively translating ribosomes. Fractions containing the small (28S) subunit, large (39S) subunit, and fully assembled (55S) ribosome are shaded cyan, pink, and yellow, respectively. The relative abundance of a given rRNA in each fraction was calculated as the percentage relative to the total RNA abundance in all fractions after normalizing to a luciferase control RNA that was spiked into each of the fractions prior to RNA isolation. Control = *UAS-mCherry-RNAi* driven by *elav-GAL4* and *da-GAL4*. *Lon*^*KD*^ = *UAS-Lon-RNAi-1* driven by *elav-GAL4* and *da-GAL4*

To investigate the mechanism by which inactivation of Lon impairs mitochondrial translation, we assessed the state of mitochondrial ribosome assembly by performing sucrose density gradient sedimentation analyses. Mitochondrial extracts from *Lon*^*KD*^ flies and controls were subjected to sucrose density gradient analysis as previously described^30^. Fractions from the sucrose gradient were then subjected to qRT-PCR to quantify the distribution of the *12S* and *16S* mitochondrial ribosomal RNAs, which mark the small (28S) and large (39S) mitochondrial ribosomal subunits, respectively. Co-localization of the *12S* and *16S* mitochondrial ribosomal RNAs within the same fraction is diagnostic for fully assembled and actively translating mitochondrial ribosomes (55S). This analysis revealed that the abundance of *12S* and *16S* ribosomal RNAs in fully assembled mitochondrial ribosome fractions (fraction 15–17) was decreased by 5.8% and 6.4%, respectively, in *Lon*^*KD*^ flies relative to the controls (Fig. 4).

**Fig. 5:**
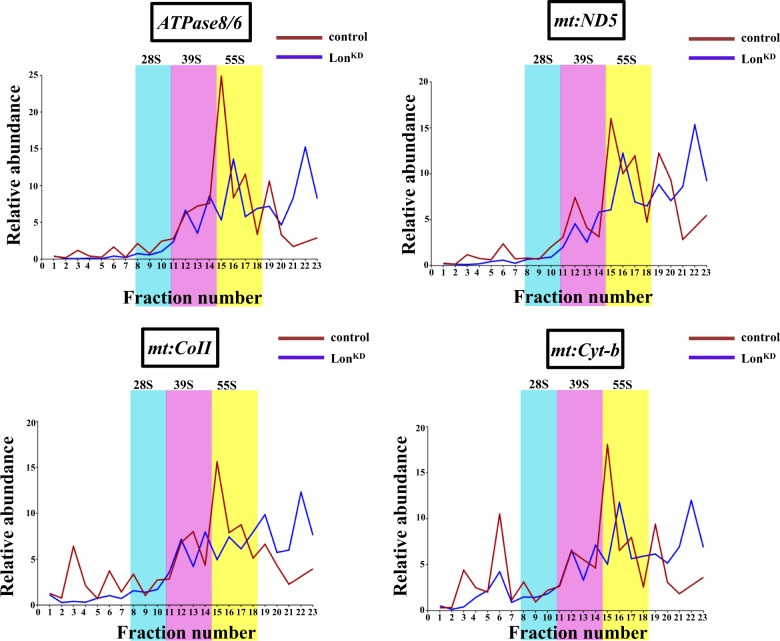
Mitochondrial RNAs accumulate in large untranslated particles upon *Lon* knockdown. qRT-PCR analysis of individual sucrose gradient fractions was used to characterize the distribution of the indicated mtDNA-encoded mRNAs relative to the 28S and 39S subunits, and fully assembled 55S mitochondrial ribosomes. Mitochondrial homogenates for sucrose density fractionation were prepared from 21-day-old adult *Lon*^*KD*^ and control flies. Relative abundance represents the fraction of the mRNA in any given fraction relative to the total after normalizing to a luciferase control RNA that was added to each of the fractions prior to RNA isolation. Control = *UAS-mCherry-RNAi* driven by *elav-GAL4* and *da-GAL4*. *Lon*^*KD*^ = *UAS-Lon-RNAi-1* driven by *elav-GAL4* and *da-GAL4*

The mild decrease in mitochondrial ribosome assembly appeared insufficient to account for the translational defect of *Lon*^*KD*^ flies, especially in light of the fact that mitochondrial transcripts are elevated in abundance relative to the controls. Thus, we examined other possible explanations for the translational defect of Lon-deficient flies. One possible explanation of our findings was that mitochondrial transcripts are translated at lower efficiency in *Lon*^*KD*^ flies. To test this model, we used sucrose density gradient centrifugation to quantify the relative distribution of four different mitochondrial transcripts, including *mt:ND5*, *mt:Cyt-b*, *mt:CoII*, and *ATPase8/6*, in gradient fractions (Fig. 5). All of these mRNAs displayed a predominant sedimentation peak co-migrating with the 55S ribosome in control samples. By contrast, in *Lon*^*KD*^ flies a smaller proportion of these transcripts co-migrated with the 55S ribosome, and this decrease was accompanied by a striking increase in the proportion of these transcripts that sedimented to the bottom of the gradient (Fig. 5). This finding suggests that a substantial proportion of the mitochondrial transcripts in *Lon*^*KD*^ flies are packaged into large untranslated particles.

### *Lon* knockdown flies accumulate unfolded mitochondrial proteins and trigger the mitochondrial unfolded protein response

Lon is believed to play a critical role in the degradation of unfolded mitochondrial proteins^34–36^. The accumulation of unfolded proteins in multiple cellular compartments has been shown to activate a stress response known as the unfolded protein response (UPR) that is specific to the compartment in which the unfolded proteins reside^37–39^. The cytosolic and endoplasmic reticulum (ER) UPR restore protein homeostasis through a two-tiered system consisting of increased expression of chaperones to facilitate the refolding of misfolded proteins, and phosphorylation-mediated inactivation of the cytosolic translation-initiation factor eIF2α to attenuate translation^40^. While previous work has established that unfolded protein stress in the mitochondria triggers the induction of chaperones and proteases, whether mitochondrial translation is inhibited by unfolded protein stress is less clear. Our findings that Lon inactivation appears to result in the sequestration of mitochondrially encoded mRNAs into translationally inactive particles, coupled with the severe defect in mitochondrial translation, led us to hypothesize that the mitochondrial UPR triggers translational inhibition.

**Fig. 6:**
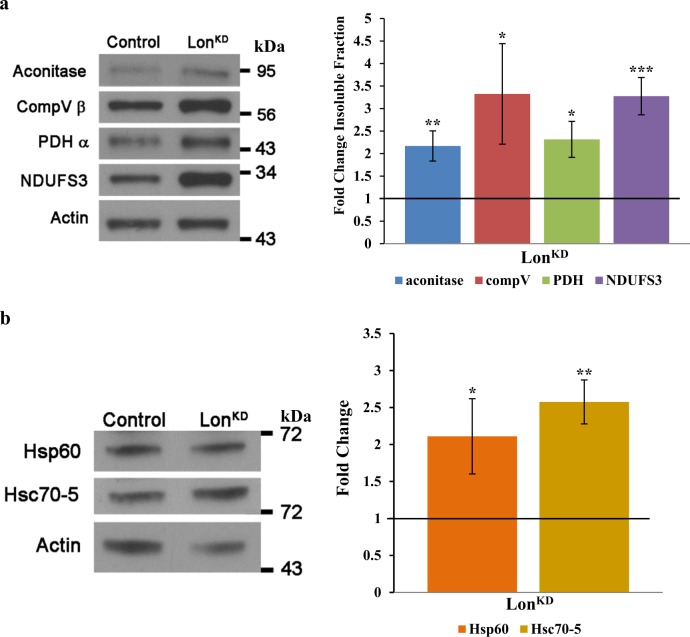
Inactivation of Lon protease results in the accumulation of unfolded mitochondrial proteins. **a** Triton-insoluble mitochondrial proteins detected by western blot in heads from 1-day-old control and *Lon*^*KD*^ flies, using antibodies to complex Vβ, PDHα, aconitase, and NDUFS3. The results were quantified by ratio to actin and normalized to control levels. The experiment was repeated at least three times. *p *<* 0.05, **p *<* 0.005, ***p *<* 0.0005 by Student’s *t*-test. **b** Immunoblot analysis of head proteins using antibodies to Hsp60A and Hsc70-5. Protein was extracted from heads of day 1 control and *Lon*^*KD*^ flies using RIPA buffer. Quantification was performed as in (**a**). Experiments were repeated at least three times. *p *<* 0.05, **p *<* 0.005 by Student’s *t*-test. Control = *UAS-mCherry-RNAi* driven by *elav-GAL4* and *da-GAL4*. *Lon*^*KD*^ = *UAS-Lon-RNAi-1* driven by *elav-GAL4* and *da-GAL4*

To explore our hypothesis we first analyzed whether *Lon*^*KD*^ flies accumulate unfolded mitochondrial proteins and whether they activate the mitochondrial UPR. To test whether unfolded mitochondrial proteins accumulate in *Lon*^*KD*^ flies, we prepared fly head protein extracts using Triton X-100. We then subjected Triton-soluble and -insoluble proteins to western blot analysis using antisera to selected mitochondrial proteins to quantify the proportion of each protein in the soluble and insoluble fractions. *Lon*^*KD*^ flies had higher levels of insoluble mitochondrial proteins than the control flies at both day 1 and day 21 of age (Fig. 6a and Suppl. Figure 6a), indicating that *Lon*^*KD*^ flies accumulate unfolded mitochondrial proteins. We next examined the abundance of the mitochondrial UPR markers Hsp60 and Hsc70-5 in *Lon*^*KD*^ flies. This analysis revealed a marked increase in both Hsp60 and Hsc70-5 in *Lon*^*KD*^ flies at day 1 and at day 21 of age (Fig. 6b and Suppl. Figure 6b). Together, these findings indicate that *Lon*^*KD*^ flies accumulate unfolded proteins and trigger the mitochondrial UPR.

### Overexpression of ClpP protease ameliorates the defect of *Lon* knockdown flies

**Fig. 7:**
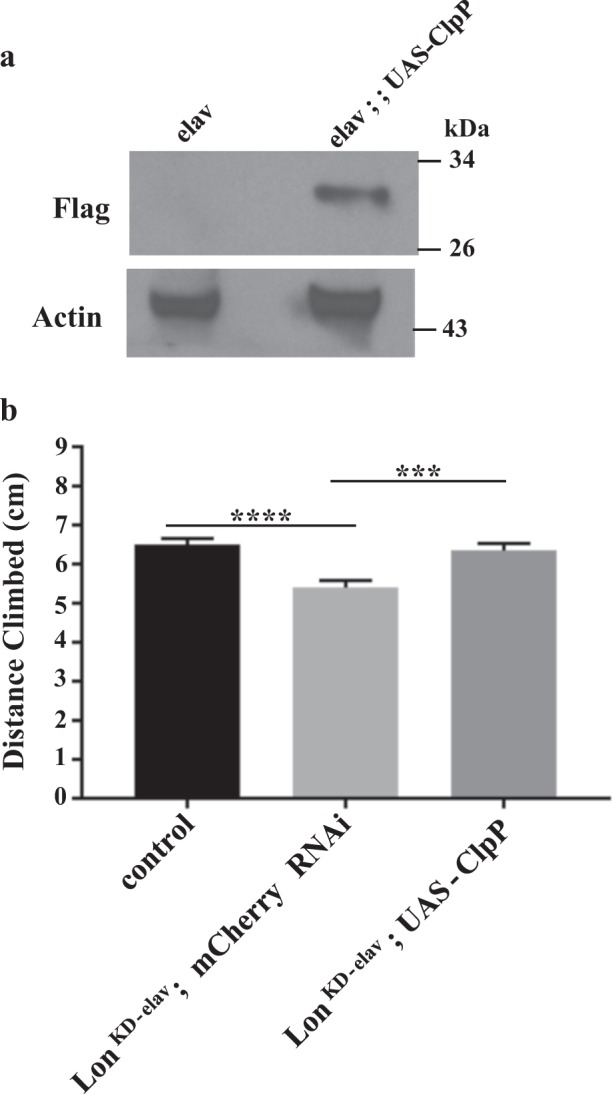
ClpP overexpression rescues the climbing defect of Lon-deficient flies. **a** Immunoblot analysis of fly heads to confirm expression of FLAG-tagged ClpP in flies bearing a *UAS-ClpP-FLAG-HA* transgene and the *elav-GAL4* pan-neuronal driver. **b** Climbing was measured in 1-day-old control flies (*n* *=* 94), *Lon*^*KD-elav*^ flies (*n* = 91), and *Lon*^*KD-elav*^ flies co-expressing ClpP protease (*n* = 83). Control = *UAS-mCherry-RNAi* driven by *elav-GAL4*. *Lon*^*KD-elav*^*; mCherry RNAi* *=* *UAS-Lon-RNAi-1* and *UAS-mCherry-RNAi* driven by *elav-GAL4*. *Lon*^*KD-elav*^*; UAS-ClpP* = *UAS-Lon-RNAi-1* and *UAS-ClpP-FLAG-HA* driven by *elav-GAL4*. Error bars represent SEM. ***p *<* 0.0005, ****p *<* 0.0001 by Student’s *t*-test

Protein quality control in the mitochondrial matrix is regulated by an elaborate network of proteases and chaperones, including the AAA^+^ protease family member Clp protease. To test whether the accumulation of unfolded proteins in *Lon*^*KD*^ flies is responsible for the phenotypes associated with Lon inactivation, we co-expressed the proteolytic subunit of the *Drosophila* Clp protease (*CG5045*, hereafter ClpP) along with the *Lon-RNAi-1* RNAi construct and examined whether ClpP overexpression is capable of rescuing the locomotor defect of *Lon*^*KD*^ flies. To account for possible titration of GAL4 protein in the presence of two UAS transgenes, we compared *Lon* knockdown flies overexpressing ClpP to *Lon* knockdown flies expressing mCherry RNAi. However, strong overexpression of ClpP in *Lon*^*KD*^ flies worsened the locomotor defect (Suppl. Figure 7). Thus, we tested whether ClpP expression could rescue a climbing defect caused by expressing the *Lon-RNAi-1* transgene using just the *elav-GAL4* driver (designated as *Lon*^*KD-elav*^). We first confirmed that driving the *UAS-ClpP* transgene with *elav-GAL4* resulted in the production of detectable ClpP protein (Fig. 7a) and use of the *elav-GAL4* driver in conjunction with the *Lon-RNAi-1* transgene reduced Lon expression (Suppl. Figure 8) and caused a climbing defect (Fig. 7b). Results of this analysis revealed that ClpP overexpression rescued the locomotor defect of *Lon* knockdown flies, thus supporting the hypothesis that the accumulation of unfolded proteins is responsible for the phenotypes of Lon-deficient flies (Fig. 7b).

## Discussion

Mitochondria are particularly prone to damage. They are the primary source and recipient of damaging ROS and continual replication of the mitochondrial genome throughout life culminates in mutation frequencies often orders of magnitude greater than that of the nuclear genome^4^. Moreover, expression of the mitochondrial and nuclear genomes must be coordinated to ensure proper stoichiometry of mitochondrial and nuclear-encoded respiratory chain components. An imbalance in this coordination can result in the accumulation of misfolded proteins and mitochondrial dysfunction^39^. The AAA^+^ mitochondrial protease family is believed to play an important role in maintaining mitochondrial competence by degrading and thereby facilitating the replacement of oxidatively damaged and misfolded proteins. However, we know little of the cellular responses and the pathogenic mechanisms of diseases caused by mutations in the genes that encode the AAA^+^ proteases. Our current work advances our understanding of these matters by showing that inactivation of the AAA^+^ protease Lon results in respiratory chain defects that appear to result from translational inhibition. Our findings further indicate that the translational defect caused by Lon inactivation results at least in part from the sequestration of mtDNA-encoded mRNAs into translationally inactive particles. Finally, we find that insoluble mitochondrial matrix proteins accumulate in *Lon*^*KD*^ animals, suggesting that mitochondrial protein aggregation triggers translational inactivation as a stress response. Our work provides a foundation to further explore the mechanisms by which mitochondrial unfolded protein stress inhibits mitochondrial translation.

Protein unfolding in the ER and cytoplasm induce well-characterized stress responses^37,38,40–43^. Each of these stress pathways has two arms: increased expression of chaperones to refold proteins, and translational inhibition to limit further protein synthesis. Translational inhibition is mediated by phosphorylation of eIF2α and culminates in the formation of translationally stalled ribonucleoprotein/mRNA complexes known as stress granules^44^. More recently, work in a variety of experimental systems has established the existence of a mitochondria-specific version of the unfolded protein response (UPR^mt^)^45–49^. Although the degree to which this pathway is evolutionarily conserved remains an active area of investigation, it has been shown in both vertebrate and invertebrate models that mitochondrial unfolded protein stress triggers the transcriptional activation of genes involved in protein folding and degradation^45,47^. However, previous work has not clearly established whether the UPR^mt^ also results in inhibition of mitochondrial translation. Recently published findings in vertebrate cell culture have revealed that inactivation of the matrix chaperone Trap1 results in inhibition of mitochondrial translation as a consequence of a pre-RNA processing defect^50^. Our findings that *Lon* knockdown flies have both an excess of mitochondrial unfolded proteins and an impairment of mitochondrial translation raise the possibility that the UPR^mt^, like other unfolded protein stress pathways, has a second arm involving translational inhibition. However, the precise mechanism by which mitochondrial unfolded protein stress in Lon-deficient animals inhibits mitochondrial translation will require further investigation.

Although most of the respiratory chain complexes exhibited reduced activity and abundance upon Lon inactivation, complex II activity was increased. This increase may simply represent a compensatory response, as complex II has no mitochondrially encoded subunits, and increased complex II activity has previously been observed in mutants with defects in mitochondrial gene expression^30,51,52^. However, it is also possible that increased complex II activity is a consequence of reduced degradation of the complex II assembly factor Sdh5, given that previous work has shown that Lon mediates the turnover of Sdh5^53^. *Lon* knockdown might thus reduce the degradation of Sdh5 and promote complex II assembly. The increased abundance of mitochondrially encoded transcripts upon Lon inactivation may likewise reflect either a general compensatory response or a specific change due to altered turnover of a possible Lon substrate. Increased mitochondrial transcript abundance has been reported previously in mutants with defective mitochondrial RNA processing and translation, suggesting that increasing the abundance of mitochondrial mRNA is a common response to inadequate mitochondrial translation^30,31,52^. However, the accumulation of mitochondrially encoded RNAs in *Lon*^*KD*^ flies could also be explained by the finding that these flies accumulate the RNA-binding protein Lrpprc1 (leucine-rich pentatricopeptide repeat motif-containing 1 protein; Suppl. Figure 9), which is known to stabilize mitochondrial transcripts^51,54,55^. Finally, Lon is a known component of mitochondrial nucleoids, and could therefore influence the turnover and abundance of other proteins involved in the synthesis or stabilization of mitochondrial transcripts^56^. Future work will be required to address these matters.

There are many unanswered questions regarding the biological roles of the AAA^+^ protease family and the mitochondrial response to unfolded protein stress. For example, mutations in the human gene encoding Lon cause a developmental disorder known as CODAS syndrome^23^. However, the underlying mechanisms causing disease are completely unknown. Although Lon is well known to promote the degradation of oxidatively damaged and unfolded proteins, the specific protein substrates of Lon are largely unknown. Whether unfolded protein stress in the mitochondria generally triggers translation inhibition, or this feature is specific to Lon inactivation is also unclear. Our study provides a foundation to address these questions and the mechanisms underlying CODAS syndrome in future work.

## Materials and methods

### Fly stocks and maintenance

*Drosophila* stocks were maintained on cornmeal-molasses food at 25 °C on a 12 h:12 h light–dark cycle. The *UAS-Lon-RNAi-1* construct *P{GD14030}-v36036* was obtained from the Vienna Drosophila Resource Center. The *w*^*1118*^, *UAS*-*mCherry RNAi* (*P{VALIUM20-mCherry}attP2*), *UAS-Lon-RNAi-2* (*P{TRiP.HMS01060}attP2), elav-GAL4*, and *da-GAL4* driver lines were obtained from the Bloomington Stock Center (Bloomington, IN, USA). The *UAS-ClpP-FLAG-HA* transgenic line was obtained from the Fly Facility, National Centre for Biological Sciences, Bangalore, India.

The *Lon* knockout allele was created using CRISPR/Cas9-mediated gene editing according to a published procedure^25,57^. Briefly, we replaced the *Lon (CG8798)* coding sequence with *DsRed* through homology-mediated repair. The following primer sequences were used for guide RNAs targeting the 5′ and 3′ UTR regions of *Lon*:

5′-Guide RNA

Sense oligo: 5′-CTTCGATAATCACTCACCACACATT-3′

Antisense oligo: 5′-AAACAATGTGTGGTGAGTGATTATC-3′

3′-Guide RNA

Sense oligo: 5′-CTTCGGGGTGTTGCGGGTGTTGAT-3′

Antisense oligo: 5′-AAACATCAACACCCGCAACACCCC-3′

Sequences flanking the *Lon* coding region were amplified from genomic DNA to facilitate homology-directed repair using the following primer sequences:

5′-Homology arm

Forward: 5′-CCGGCACCTGCGGCCTCGCAGTGCTCCGATCACGTTGGGAATGGG-3′

Reverse: 5′-CCGGCACCTGCGGCCCTACGTGTGGTGAGTGATTATGTGACGGCTGGTG-3′

3′-Homology arm

Forward: 5′-GGCCGCTCTTCATATGATAGGTTTTATAAATATCTATCGTTATCAGG-3′

Reverse: 5′-CCGGGCTCTTCTGACTTTCCCCGCCTCACCGGTGGACGGCC-3′

These homology arms were then cloned into the *pHD-DsRed-attP* vector containing the eye-specific *3xP3* promoter fused with *DsRed* and the resulting construct was microinjected into Cas9-expressing embryos by a commercial service (Rainbow Transgenic Flies Inc.). Flies bearing the *Lon* deletion were identified by screening the offspring of injected adults for expression of red fluorescence in the compound eye. Flies expressing the *DsRed* marker were then further subjected to whole-genome sequencing to verify deletion of the *Lon* gene.

### Lifespan and behavioral analyses

Lifespan and behavioral analyses were performed using male flies. Age in all these experiments refers to the number of days following eclosion. Longevity assays were performed at 25 °C and involved 20 flies per vial. Flies were transferred to fresh food every 2–3 days and the number of dead flies was recorded during each transfer. Kaplan–Meier lifespan curves were generated using GraphPad Prism v5, and we used the Mantel–Cox log-rank test to determine the statistical significance of differences in survival between tested genotypes.

For climbing and flight assays, flies were anesthetized with CO_2_ and allowed to recover for at least 24 h before the experiment. Climbing behavior was assessed using the Rapid Iterative Negative Geotaxis (RING) assay at day 1 according to a previously published protocol with minor modifications^58^. In particular, height climbed was measured on still images from video recordings rather than on photographs. Briefly, 15 flies were transferred into plastic vials and these vials were then loaded onto the RING apparatus. The apparatus was tapped down to initiate the climbing response and the height climbed by each fly after 3 s was recorded. The climbing assay was repeated three times for each group.

Flight assays were performed according to a previously published protocol^59,60^. Briefly, an acetate sheet was divided into five equal parts, coated with grease and inserted into a 2-liter graduated cylinder. One-day-old flies were tapped into a funnel at the top of the cylinder and became stuck to the vacuum grease where they alighted. The number of flies that alighted in each of the five sections was counted and multiplied by the number corresponding to each section (0-4, labeled from bottom to top). The flight index was calculated by summing these values and dividing this sum by the maximum possible score (four times the number of flies used in the assay). At least 100 flies of each genotype were used for a given experiment.

### Mitochondrial respiratory chain activity assay

Mitochondrial respiratory chain activity assays were performed according to a published procedure with several minor modifications^25^. Briefly, 1000 adult flies were homogenized in isolation buffer (5 mM Tris (pH 7.4), 250 mM sucrose, and 2 mM EGTA) with 1% (w/v) fatty acid free bovine serum albumin. The lysate was subjected to centrifugation at 600 *g* for 10 min to remove cellular debris. The supernatant was then subjected to further centrifugation at 7000 *g* for 10 min to pellet mitochondria. Mitochondria were washed twice in isolation buffer, resuspended in the same buffer, flash frozen in liquid nitrogen, and stored at −80 °C. Roughly 100 µg of mitochondria was used for each assay. Complex I activity was determined spectrophotometrically by monitoring the oxidation of NADH at 340 nm using ubiquinone-1 as an electron acceptor. Nonspecific activity was determined using 10 μM rotenone and subtracted to calculate complex I-specific activity. Complex II activity was determined by monitoring the reduction of 2,6-dichlorophenolindophenol at 600 nm in the presence of succinate and decylubiquinone. Background activity was determined using 10 mM malonate and subtracted to calculate complex II-specific activity. Complex III activity was determined by monitoring the reduction of cytochrome *c* at 550 nm in a reaction mixture containing decylubiquinol and cytochrome *c*. Nonspecific activity was determined using antimycin A and subtracted to calculate complex III-specific activity. Complex IV activity assay was performed by monitoring the oxidation of reduced cytochrome *c* at 550 nm. Background activity was determined using potassium cyanide and used to calculate complex IV-specific activity. All activities were normalized to citrate synthase activity, which was determined by following the reduction of 5,5′-dithiobis (2-nitrobenzoic acid) at 412 nm in the presence of acetyl-coenzyme A and oxaloacetate.

### Total ATP determination

Total ATP was determined from whole flies according to a previously published procedure^61^. Briefly, five 21-day-old flies were homogenized in 100 μL of homogenization buffer (6 M guanidine HCL, 100 mM Tris (pH 7.8), and 4 mM EDTA). The samples were boiled for 5 min and subjected to centrifugation for 3 min at 21,000 *g* to remove debris. The supernatant was then diluted to 1:750 and total ATP content was measured using an ATP determination kit (A22066, Molecular Probes). Total ATP content was determined by comparing the luminescence measurements for each sample to the ATP standard curve and normalized to the total number of flies used in the assay.

### Blue native PAGE (BN-PAGE) analysis and in-gel activity assay

BN-PAGE and in-gel activity assays were performed according to a previously published protocol^62^. Briefly, 100 μg of mitochondria prepared from 3-week-old adult flies was solubilized in a buffer containing a digitonin/protein (w/w) ratio of 8 and subjected to centrifugation at 20,000 *g* for 10 min at 4 °C. Coomassie G-250 was added to the supernatant and the sample was analyzed by native PAGE. Following electrophoresis, the resulting gel was subjected to in-gel activity assays as described below.

The combined complex I and IV in-gel activity assay was performed by first incubating the gel in a solution containing 1 mg/ml of the complex IV substrate cytochrome *c* along with 0.5 mg/ml 3,3′-diaminobenzidine and 45 mM phosphate buffer (pH 7.4) for 40 min. After the appearance of brown reaction products, the gel was washed with water and incubated in a solution containing 0.1 mg/ml of the complex I substrate NADH along with 2 mM Tris (pH 7.4) and 2.5 mg/ml nitrotetrazolium blue chloride for 20 min. The reaction was quenched with 10% acetic acid upon the appearance of the violet color indicative of complex I activity.

The complex II in-gel activity assay was performed by incubating the gel in a solution consisting of 5 mM Tris (pH 7.4), 20 mM sodium succinate, 2.5 mg/ml nitrotetrazolium blue chloride, and 0.2 mM phenazine methosulfate for 40 min. The reaction was quenched with 10% acetic acid upon the appearance of the violet color indicative of complex II activity.

The complex V in-gel activity assay was carried out by incubating the gel in a solution containing 35 mM Tris, 270 mM glycine, 14 mM magnesium sulfate, 10 mM adenosine triphosphate, and 0.2% lead (II) nitrate for 16 h. The reaction was stopped using 50% methanol upon the appearance of silver bands indicative of complex V activity.

### Immunoblotting

Fly heads were homogenized in RIPA buffer with protease inhibitor cocktail (Roche) for 20 min on ice, and after centrifugation at 21,000 × *g* for 20 min the supernatant was collected and subjected to western blot analysis. The antibodies used were as follows: rabbit anti-LONP1 1:500 (NBP1-81734, Novus Biologicals); mouse anti-Actin 1:50,000 (MAB1501, Chemicon/Bioscience Research Reagents); mouse anti-FLAG 1:1000 (F3165, Sigma); rabbit anti-citrate synthase 1:1000 (CISY11-A, Alpha Diagnostics), rabbit anti-Hsp60 1:500 (D307, Cell Signaling Technology); and rabbit anti-GRP 75 (H155) 1:1000 (sc-13967, Santa Cruz) in Phosphate buffered saline with 0.1% Tween-20^24^. The secondary antibodies (anti-mouse HRP and anti-rabbit HRP (Sigma)) were used at a dilution of 1:10,000. Western blot images were quantified using ImageJ software (NIH) and normalized to actin. Each experiment was performed in triplicate.

To analyze unfolded mitochondrial proteins, protein was extracted from fly heads as previously described, except that 0.5% rather than 1% Triton X-100 was used in accordance with other work on mitochondrial proteins^63,64^. Antibodies for assaying mitochondrial protein folding status were used as follows: mouse anti-NDUFS3 1:500 (ab14711, Abcam), mouse anti-Complex V Beta 1:2000 (A-21351, Invitrogen), mouse anti-PDH E1α (clone 8D10E6) 1:1000 (45-660-0, Fisher Scientific), and rabbit anti-aconitase (ACO2) 1:2000 (AP1936C, Abgent).

### Genomic DNA isolation and mitochondrial DNA copy number estimation

Mitochondrial and nuclear DNA was isolated from 40–50 heads obtained from 21-day-old adult flies using the DNeasy Blood & Tissue kit (Qiagen). A total 5 ng of genomic DNA was used as a template to perform qPCR using iTaq Universal SYBR Green Supermix (Bio-Rad). Mitochondrial DNA levels were estimated by using primers (Supplemental Table 1) to amplify the *mt:Cyt-b* gene, and normalized to levels of the nuclear gene *Act79b*. The relative fold change was determined through the 2^−ΔΔCt^ method^30^.

### RNA isolation and quantitative reverse transcription-PCR (qRT-PCR)

RNA isolation and qRT-PCR was performed according to a previously published procedure^24,30,51^. Total RNA was isolated from 40–50 heads obtained from 21-day-old adult flies using the Direct-zol RNA MiniPrep kit (Zymo Research). The RNA was reverse transcribed to cDNA using the iScript cDNA Synthesis Kit (Bio-Rad). For RNA quantification, qRT-PCR experiments were performed using iTaq Universal SYBR Green Supermix and a LightCycler 480 (Roche). Each sample was analyzed in triplicate and normalized to *Act79b* transcript abundance. The relative fold change was determined by the 2^−ΔΔCt^ method. All primers used for qRT-PCR are listed in Supplemental Table 1.

### *In organello* translation

*De novo* labeling of mitochondrial translation products was performed as previously described^30,51^. Approximately 750 µg of mitochondria isolated from third instar larvae or adult flies was resuspended in 500 µl of translation buffer (100 mM mannitol, 10 mM sodium succinate, 80 mM potassium chloride, 5 mM magnesium chloride, 1 mM potassium phosphate, 25 mM HEPES (pH 7.4), 60 μg/ml all amino acids except methionine, 5 mM ATP, 0.2 mM GTP, 6 mM creatine phosphate, and 60 μg/mL creatine kinase) supplemented with 500 μCi/ml of ^35^S-methionine (Perkin–Elmer). Following incubation at 30 °C for 1 h, mitochondria were washed four times using isolation buffer and resuspended in SDS sample buffer. Roughly 300 µg of mitochondria was subjected to SDS-PAGE. Following electrophoresis, the gel was dried and exposed to a phosphor screen. The phosphor screen was scanned using a gel imaging scanner (GE Typhoon FLA 9000). Mitochondrial translation profile was compared to a previously published study done in *Drosophila* larvae^30^. Roughly 75 µg of mitochondria was loaded on gel and stained with Coomassie to use as a loading control.

### Mitochondrial ribosomal profiling using sucrose density gradient assay

Mitochondrial ribosomal profiling was performed according to a previously published procedure with minor modifications^30^. Freshly isolated mitochondria (2 mg) were incubated on ice in lysis buffer (260 mM sucrose, 100 mM NH_4_Cl, 10 mM MgCl_2_, 30 mM Tris-HCl pH 7.5, 40 U/ml Protector RNase Inhibitor, and 1% Triton X-100) supplemented with EDTA-free complete protease inhibitor cocktail (Roche) and PhosSTOP phosphatase inhibitor cocktail (Roche). Mitochondrial lysates were cleared by pelleting the debris through centrifugation at 10,000 *g* for 45 min at 4 °C. The supernatant was then loaded onto a 7–47% linear sucrose gradient made in a buffer containing 100 mM NH_4_Cl, 10 mM MgCl_2_, 30 mM Tris-HCl pH 7.5, and EDTA-free complete protease inhibitor cocktail (Roche). Samples were then subjected to centrifugation at 39,000 rpm for 6 h at 4 °C. Fractions of 500 μl from the sucrose gradient sedimentation were collected and RNA was extracted from each fraction using TRIzol LS Reagent (Invitrogen) and the Direct-zol RNA MiniPrep kit. RNA was reverse transcribed and qRT-PCR analyses were performed to quantify RNAs as described above.

### Statistics

All findings are presented as mean ± SEM. Unless otherwise stated, statistical significance was calculated using two-tailed Student’s *t*-test in GraphPad Prism v5.

## Electronic supplementary material

Supplemental Figures

SUPPLEMENTAL TABLE
