# Supplementary material for: Lon protease inactivation in Drosophila causes unfolded protein stress and inhibition of mitochondrial translation
Source: Cell Death Discov. 2018 Oct 22;4:51. doi: 10.1038/s41420-018-0110-1 (PMC6197249; doi:10.1038/s41420-018-0110-1)
Supplement: Supplementary file 1 — Supplemental Figures [file 41420_2018_110_MOESM1_ESM.docx]

**
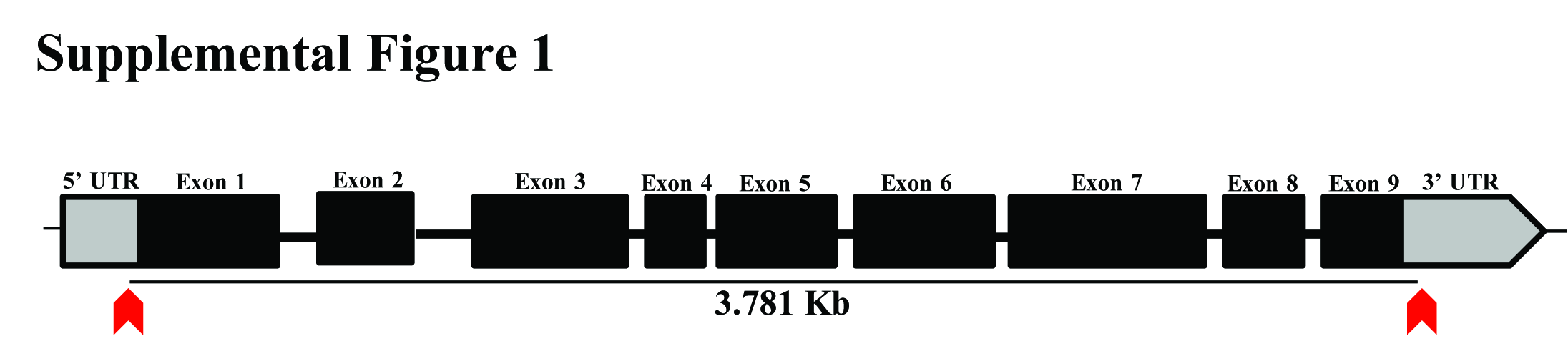
**

**Supplemental Figure S1**: Lon in *Drosophila* is encoded by the *Lon protease* (*CG8798)* gene, which consists of 9 exons (black boxes). Red arrowheads highlight the region targeted by guide RNAs for CRISPR-mediated deletion of the *Lon* gene.

**
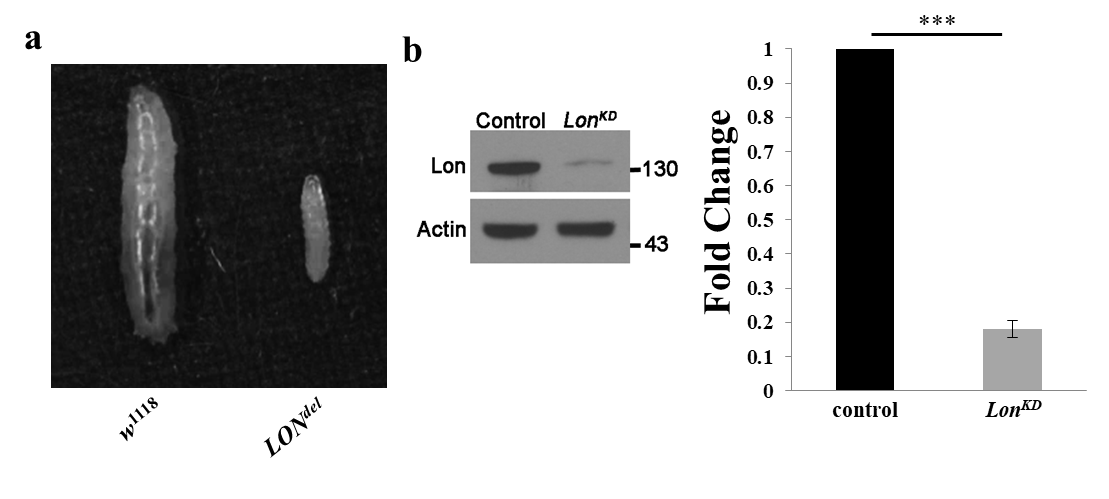
**

**Supplemental Figure S2**: (a) Size comparison of wild-type control (WT) and isogenic *Lon* knockout larvae 96 hours after hatching. (b) Immunoblot analysis from whole bodies of 1-day-old control and *Lon^KD^* flies using Lon and actin antibodies. The band intensity is normalized against actin as a loading control. The experiment was repeated at least three times. Significance was determined using Student's t-test (****p* < 0.0005)**.**

**
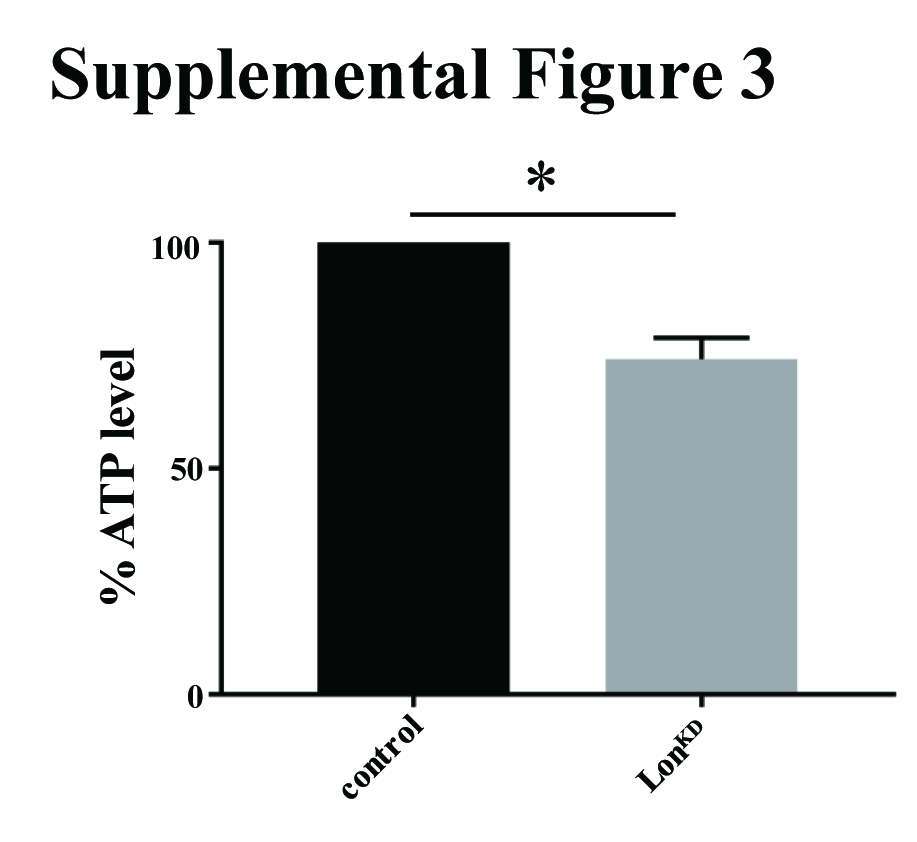
**

**Supplemental Figure S3**: Relative change in ATP levels in 21-day-old control and *Lon^KD^* flies (*n* = 4 independent groups of 5 flies). Significance was determined using Student's t-test, **p* < 0.05.

**
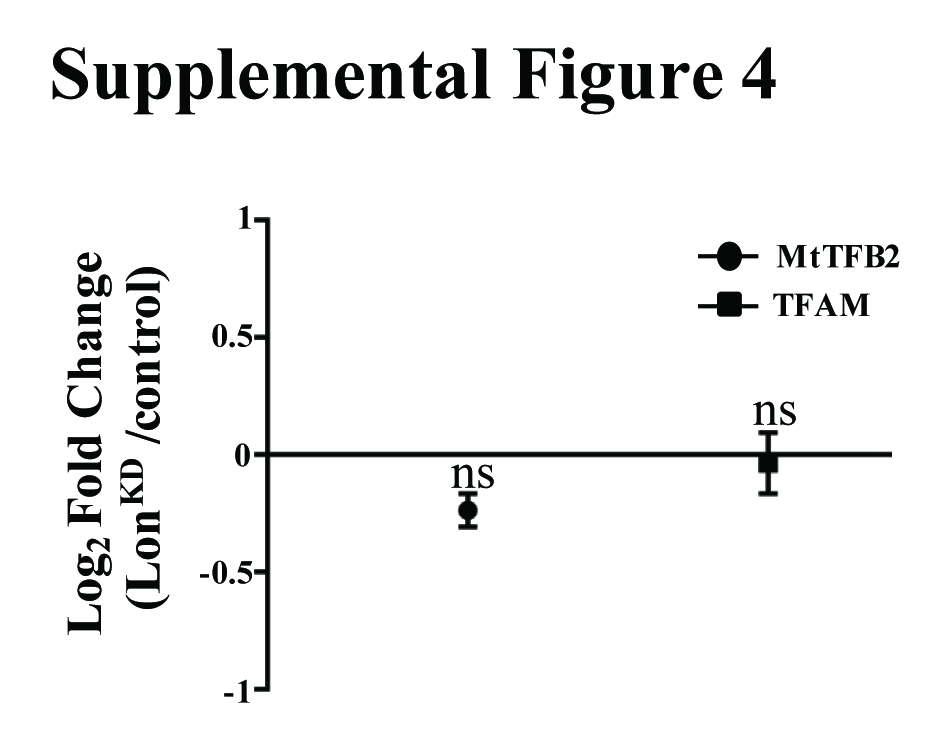
**

**Supplemental Figure S4**: Relative log_2_ fold change in steady-state levels of *TFAM* and *mtTFB2* mRNA from 21-day-old adult fly heads, measured by qRT-PCR and normalized to levels of the nuclear gene *Act79b* (*n* = 3 independent groups of 40-45 fly heads). The display indicates mean ± SEM. Student's t-test was applied; ns = non-significant.

**Control**

**26**

**34**

**43**

**55**

**72**

**96**

**10**

**kDa**

**17**


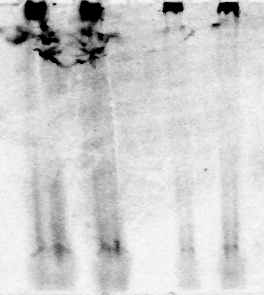


**Supplemental Figure S5**: *In organello* translation was performed using mitochondria isolated from 21-day-old control flies. Mitochondria were labeled by incubating with ^35^S-methionine for 1 hour. Position of protein molecular weight marker is indicated.

**
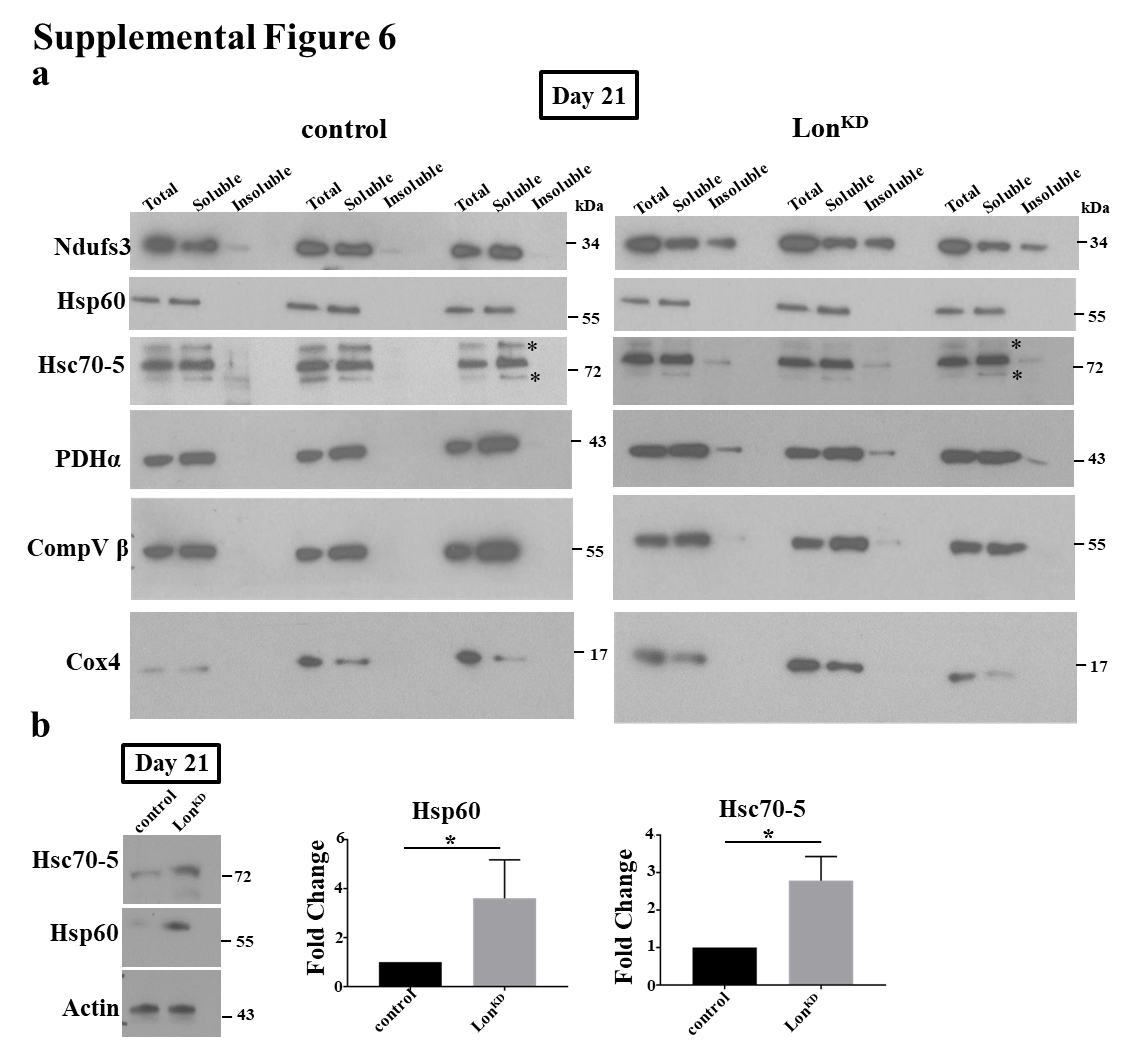
**

**Supplemental Figure S6**: **(a)** Triton X-100 soluble and insoluble mitochondrial proteins were detected by western blot in heads from 21-day-old control and *Lon^KD^* flies, using antibodies to NDUFS3, Hsp60, Hsc70-5, ATPsynβ, PDHα (PDH E1α) and Cox4. Asterisk indicates nonspecific band; *n* = 3 independent groups of 40-45 fly heads. **(b)** Immunoblot analysis of heads from day 21 control and *Lon^KD^* flies using antibodies to Hsp60 and Hsc70-5 (*n* = 3 independent groups of 40-45 fly heads).

**
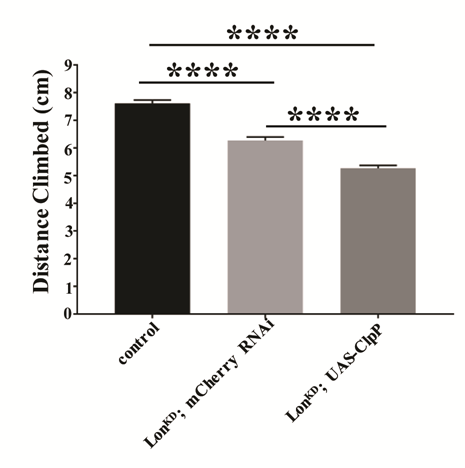
**

**Supplemental Figure S7**: Climbing was measured in 1-day-old control flies (*n* = 90), *Lon^KD^* flies (*n* = 90) co-expressing the *UAS-mCherry RNAi*, and *Lon^KD^* flies co-expressing the *ClpP* protease (*n* = 100). Both *elav-GAL4* and *da-GAL4* were present in all animals. Error bars represent SEM. *****p* < 0.0001 by Student’s t-test.

**
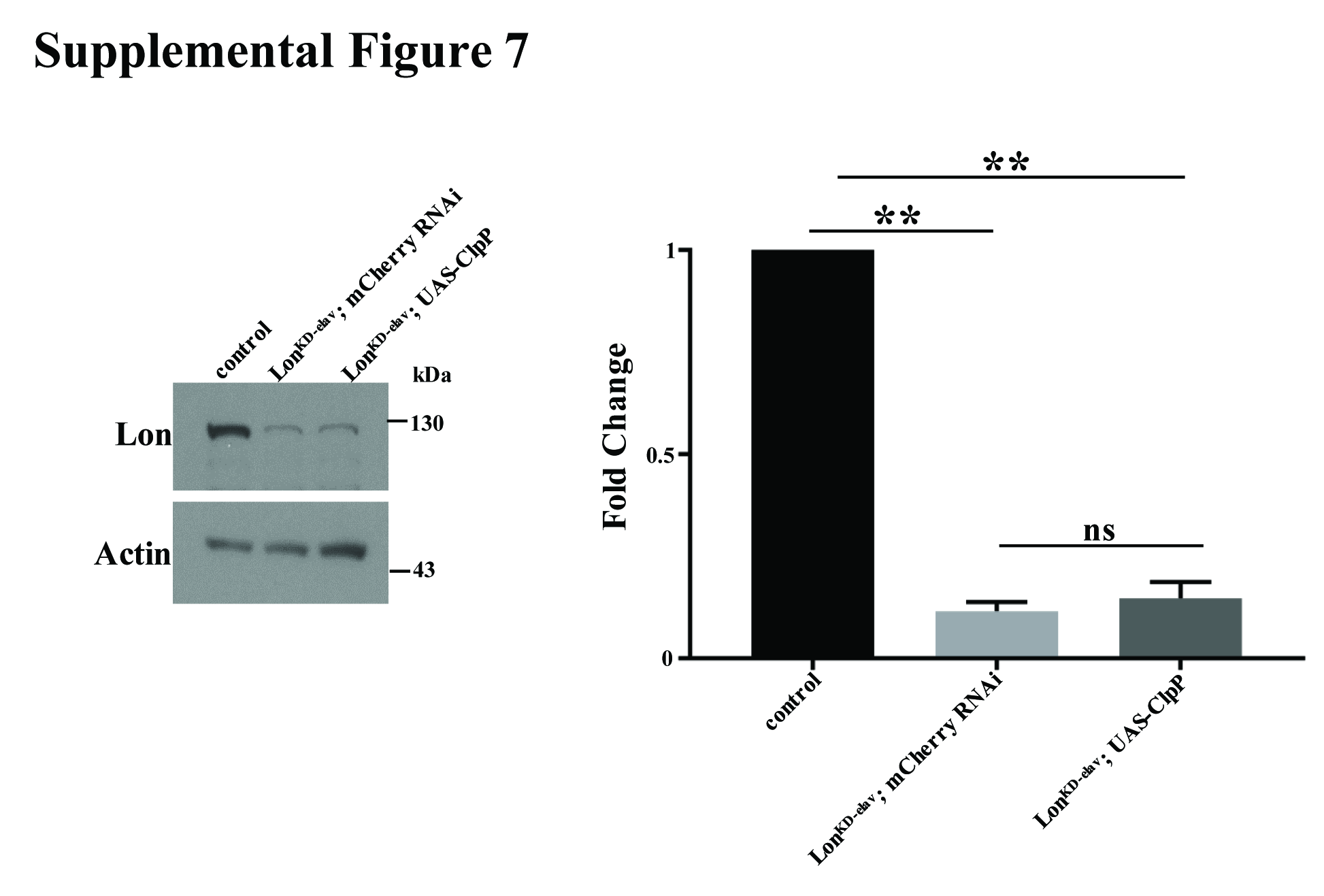
**

**Supplemental Figure S8**: Immunoblot analysis from 1-day-old control flies, *Lon^KD-elav^* flies co-expressing the *UAS-mCherry RNAi*, *Lon^KD-elav^* flies co-expressing the ClpP protease using Lon and actin antibodies. Note that only one driver (*elav-GAL4*) was present. The band intensity was normalized against actin as a loading control. *n* = 3 independent groups of 15 fly heads. Significance was determined using Student's t-test, ns = non-significant, ***p* < 0.005. The presence of the *UAS-ClpP* transgene did not attenuate Lon expression relative to flies bearing the mCherry RNAi construct.

**
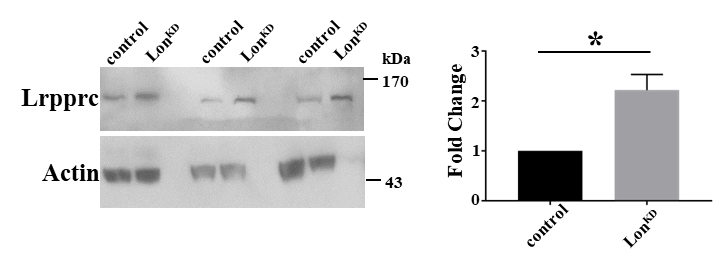
**

**Supplemental Figure S9**: Immunoblot analysis from 21-day-old control and *Lon^KD^* flies using antisera against Lrpprc1 and actin. *n* = 3 independent groups of 15 fly heads. The band intensity was normalized against actin as a loading control. Significance was determined using Student's t-test, **p* < 0.05.
