# Supplementary material for: Lon protease inactivation in Drosophila causes unfolded protein stress and inhibition of mitochondrial translation
Source: Cell Death Discov. 2018 Oct 22;4:51. doi: 10.1038/s41420-018-0110-1 (PMC6197249; doi:10.1038/s41420-018-0110-1)
Supplement: Supplementary file 2 — SUPPLEMENTAL TABLE [file 41420_2018_110_MOESM2_ESM.docx]

| 12SrRNA For | CAGATCAAGGTGTAGCTTATATTTAAGTAA |
| --- | --- |
| 12SrRNA rev | GAGCGACGGGCGATGTGTACATA |
| 16SrRNA For | GATTGCGACCTCGATGTTGGATTAAGAT |
| 16SrRNA rev | GAAACCAACCTGGCTTACACCGGT TTG |
| CYTOCHROME B For | GGATACGTATTACCTTGAGGACAAATAT |
| CYTOCHROME B rev | GTGGCATTATCAACAGCAAATCCACCTCAT |
| COX1 For | CCTCAGCTACTATAATTATTGCAGTTCC |
| COX1 Rev | CAACTCCTGTTAATCCTCCTACTGTA |
| COX2 For | GAATCGGCCATCAATGATATTGAAGTTACG |
| COX2 Rev | GTTCAAGAATGAATAACATCAGCAGCTG |
| COX3 For | CCATTAACAGGAGCTA TCGGAGC |
| COX3 Rev | CCTTCTCGTGATACATCTCGTCATCAT |
| ND1 For | AAGGAGTCCGATTAGTTTCAGCTAATG |
| ND1 Rev | GGAGGTTTGCGAGCTGTGGCTCAGA |
| ND5 For | GCAGAAACAGGTGTAGGAGCAGCTATAG |
| ND5 Rev | GGGGATGTAGCTTTACTTCTTTCTATTG |
| ND4 For | GCAATTAAAGCCTTTAAATCAGTTTGACG |
| ND4 Rev | CTCCAGTTTCTGGGTCTATAATTTTAGC |
| ATP6/8 For | CTGTATTCGACCCCTCAGCTATTT |
| ATP6/8 Rev | TAGATCCATTATGACCTGATGGGCC |
| ACTIN79B For | CTGGCGGCACTACCATGTATC |
| ACTIN79b Rev | GGACCGGACTCGTCATACTC |
| TFAM For | TGGAGGAGCGAACAAAGTACG |
| TFAM Rev | GACCCAAAAAGCCCGCTTC |
| MTTFB2 For | GAACTGGTGACTCGCTACAGC |
| MTTFB2 Rev | GCAGCAGCCTCCGAATTTG |
